# *Lactobacillus plantarum* strains show diversity in biofilm formation under flow conditions

**DOI:** 10.1016/j.heliyon.2022.e12602

**Published:** 2022-12-24

**Authors:** P. Rashtchi, M. Tempelaars, E. van der Linden, T. Abee, M. Habibi

**Affiliations:** a Physics and Physical Chemistry of Foods, Wageningen University, Wageningen 6708WG, the Netherlands; b Food Microbiology, Wageningen University, Wageningen 6708WG, the Netherlands

**Keywords:** Biofilm, Fluid flow, *Lactobacillus plantarum*

## Abstract

In many natural and technological applications, microbial biofilms grow under fluid flow. In this project, we investigated the influence of flow on the formation and growth of biofilms produced by gram-positive *Lactobacillus plantarum* strains WCFS1 and CIP104448. We used an in-house designed device based on a 48-well plate with culture volumes of 0.8 ml, and quantified total biofilm formation under static and flow conditions with flow rates 0.8, 1.6, 3.2 and 4.8 ml/h (with 1, 2, 4 and 6 volume changes per hour) using crystal violet (CV) staining, and determined the number of viable biofilm cells based on plate counts. The amount of total biofilm under flow conditions increased in the CIP 104448 strain, with significantly increased staining at the wall of the wells. However, in the WCFS1 strain, no significant difference in the amount of biofilm formed under flow and static conditions was observed. Plate counts showed that flow caused an increase in the number of viable biofilm cells for both strains. In addition, using enzyme treatment experiments, we found that for WCFS1 in the static condition, the amount of mature biofilm was declined after DNase I and Proteinase K treatment, while for flow conditions, the decline was only observed for DNase I treatment. The CIP104448 biofilms formed under both static and flow conditions only showed a decline in the CV staining after adding Proteinase K, indicating different contributions of extracellular DNA (eDNA) and proteinaceous matrix components to biofilm formation in the tested strains.

## 1 Introduction

Microbial biofilms result from the adhesion and growth of bacteria into a multicellular community on a surface embedded in an extracellular matrix (Toole et al. 2000). In many natural, technological and food processes applications, microbial biofilms grow under fluid flow. Biofilm formation can cause many problems in the food industry such as product spoilage, food safety problems, and loss of production efficiency (Satpathy et al., 2016). Biofilm formation is also relevant in bacteria-host interaction and has been linked to gastrointestinal tract colonization and foodborne pathogen and clinical infections (Vestby et al., 2020). Fluid flow has a considerable influence on microbial surface adhesion and colonization, including the structure and matrix composition of biofilms. The presence of flow affects the attachment, nutrient supply, colonization, and conceivably affects the efficacy of chemical signalling molecules important in quorum-sensing phenomena involved in biofilm maturation (Emge et al., 2016). Despite extensive studies on biofilm formation in static conditions, the influence of flow on the development of microbial biofilms and their composition and stability is limited. A number of approaches have been used to pursue biofilm formation in flowing conditions including the use of microfluidics, drip flow, and rotating disk reactors (Schwartz et al., 2010; Oder et al., 2018; Lee et al. 2008; Pereira et al., 2002; Rusconi et al., 2011). In most of studies, selected species of gram-negative bacteria including *Escherichia coli*, *Legionella pneumophila*, *Pseudomonas* spp., and *Burkholderia cepacia* have been studied under flow conditions (Emge et al., 2016; Mampel et al., 2006; Peck et al. 2019; Teodósio et al., 2011). Gram-positive bacteria also play a crucial role in many environmental, food processing, and medical settings, and only a limited number of studies have assessed the influence of flow on the development and growth of biofilms. Limited studies on *Bacillus subtilis*, *Listeria monocytogenes* and *Staphylococcus* spp, reveal the significant effects of dynamic medium, medium composition and ambient temperature on biofilm formation and its structure (Chang et al., 2020; Cherifi et al., 2017; Lee et al., 2008; Mendez et al., 2020). For example, *Staphylococcus epidermidis* biofilm formation in high flow conditions showed monolayers of cells, whereas, at lower flow rates, multi-layered and clump-like structures became apparent (Lee et al., 2008). Assessment of *B*. *subtilis* formation showed an effect of flow rate on the distribution of cells in the biofilm with a concomitant increase in the production of exopolysaccharides in the biofilm matrix. Further increase of hydrodynamic shear force at a high flow rate resulted in a change from a thick, more open, complex structure to a thinner and denser one (Chang et al., 2020).

*Lactobacillus* spp. are non-motile, non-spore-forming gram-positive bacteria that are widely present in food industries and can cause contamination and spoilage of foods (Kubota et al., 2008), while they are also important as starter bacteria and as probiotics, with a range of species residing in the human intestinal tract (Salas-Jara et al., 2016). Knowledge about biofilms formed by *Lactobacillus* spp. is mostly limited to static conditions and the limited number of studies on biofilm formation in flowing conditions did not address the impact of flow on biofilm formation efficacy and quantification of the number of viable biofilm cells (Kubota et al., 2008; Salas-Jara et al., 2016; Slízǒvá et al., 2015).

*Lactobacillus plantarum* is one of the most studied representatives of the genus *Lactobacillus*, and strains have been isolated from a wide range of environments including soil, plant and animal-based foods, to oral cavities and intestines of animals and humans (Aoudia et al., 2016; Kleerebezem et al., 2003; Matejčeková et al., 2016). It is shown that Brain Heart Infusion medium supplemented with manganese and glucose and incubation at 30 °C constitute optimum conditions for static biofilm formation of *L*. *plantarum* WCFS1, a well-recognized model strain isolated from human saliva (Ramírez et al., 2015). It is also shown that biofilm formation was significantly reduced in the presence of DNase I and/or proteinase K, indicating the role of extracellular DNA (eDNA) and proteins (proteinaceous material) in the biofilm maturation and matrix structure of this the strain (Ramírez et al., 2015).

In this work, we study the biofilm formation of two strains of *L. plantarum* (WCFS1and CIP104448) under flow conditions by a novel fluidic assay. We show that the flow differently affects the growth and formation of biofilms produced by the two strains of *L. plantarum*. Our results show the amount of biofilm produced by the CIP104448 strain grows by increasing the rate of flow, while the flow rate does not significantly change the biofilm formation of the WCFS1 strain. In addition, enzyme treatment shows the presence and accessibility of proteinaceous material and eDNA were different in biofilms formed under static and flow conditions. Our results reveal that the number of cells attached to the wall increases in a power-law behavior as a function of time with an exponent depending on the flow conditions.

## 2 Material and methods

### 2.1 Bacterial strains

Two strains of *Lactobacillus plantarum* (*L. plantarum* WCFS1, and *L. plantarum* CIP104448) were used in this study. *L. plantarum* WCFS1 was isolated from human saliva (Kleerebezem et al., 2003) and *L. plantarum* CIP104448 was obtained from human stool. These strains were streaked onto De Man-Rogosa-Sharpe (MRS) agar from a −80 °C stock and incubated aerobically for 48 h at 30 °C. A single colony was transferred into 10 ml of MRS, grown for 18 h at 30 °C, and used for inoculation of the biofilm experiments. For biofilm formation in static and flowing conditions, 10-fold diluted brain heart infusion (Becton, Dickinson, France) supplemented with 0.2% glucose (Merck) and 0.0005 % manganese sulphate (Merck) was used. This medium is a ten times diluted version of the medium that was used in a previous study on WCFS1 (Ramírez et al., 2015). The resultant reduction in nutrient availability, supports comparative analysis of total biofilm formation and quantification of viable biofilm cells in static and flow conditions, without and with supply of fresh medium and thus additional nutrients.

### 2.2 In-house flow system

**Figure 1:**
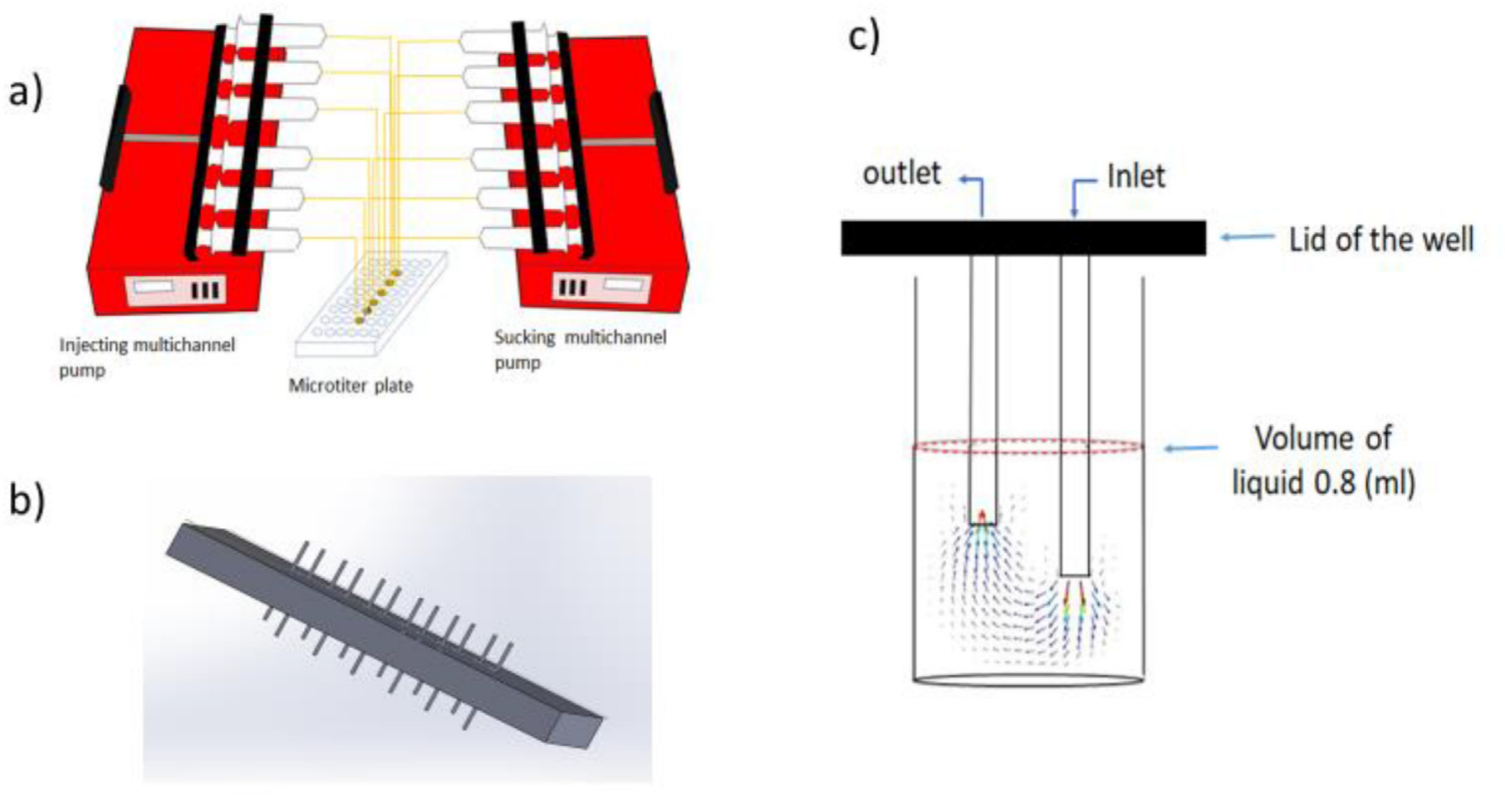
Schematic presentation of the designed set up, 3D printed lid and detail of one well. (a) Schematic presentation of the designed flow system. (b) Lid of the system designed in Solid Works and 3D printed using high-temperature resin. (c) Details of one of the wells of a 48 48-well microtiter plate, with the simulated flow field shown by a vector field.

To study biofilm formation under static and flow conditions a set-up was designed using 48- well plates, as shown in Figure 1a. The plate lid covering the wells and holding the inlet-outlet pipes was 3D printed using a high-temperature resin (Formlabs High Temp Engineering Resins) (Figure 1b). The inlet and outlet for each well were connected to two syringes via plastic tubes for injecting and removal of the culture medium. The fluidic system was first tested and calibrated by measuring the pumping and suction rates at different flow rates using water as a working fluid. Different flow rates were applied by two multi-channel programmable pumps for pumping and suction. The suction and pumping rates in both syringes were adjusted to maintain a constant amount of 0.8 ml of medium in each well resulting in a constant fluid level of 9 mm from the bottom of each well. The lid including inlet and outlet tubes was washed by distilled water and sterilized in an autoclave prior to each experiment. Next to static biofilm formation (flow rate 0 ml/h), the effect of increasing the flow rate of the medium on biofilm formation was studied by applying four different flow rates (0.8, 1.6, 3.2, and 4.8 ml/h). Based on the volume of the culture medium per well (0.8 ml), the selected flow rates resulted in 1, 2, 4 and 6 volume changes per hour, respectively. The run time of each experiment was 24 h.

### 2.3 Numerical simulation of the flow field

COMSOL Multiphysics 5.4 software was used to simulate the flow field in the fluidic system. Figure 1c shows the details of one of the wells of a microtiter plate. The diameter of the well and diameter of the inlet/outlet needles were 10 mm and 1.2 mm respectively. The height of the medium in each well is 9 mm and the distance from the inlet and outlet to the bottom of the well is 4 mm and 6 mm, respectively. A schematic view of one well is shown in Figure 1c, where the dotted line represents the level of the liquid in the well. Since the medium was a 10-fold diluted medium, the physical properties of water were chosen to simulate the flowing material with the no-slip boundary condition on the solid surfaces and open boundary for the free surface of the medium. Using these conditions, we were able to simulate the liquid velocity field and the shear rate at any point in the liquid medium. The velocity field is shown by arrows in the cross-section plane of a well in Figure 1c.

### 2.4 Biofilm analysis

#### 2.4.1 Total biofilm determination and crystal violet assay

The 48-well microtiter plates were filled with 800 μl of culture medium and inoculated with 12 μl of an overnight grown MRS broth with an OD600 of 5 containing 6.3 ± 0.3 log_10_ CFUs/ml. The initial attachment time of the bacteria to the surface of the wells was varied in two series of experiments. In one series after inoculation of the bacteria, the flow of the culture medium was applied after about 3 min due to connecting the system to the syringes, thereby conceivably excluding attachment in the static condition. In the second series, the flow was initiated after 3 h (initial attachment time set at 3 h). The system was placed in an incubator (New Brunswick™ Innova) at 30 °C, before initiating the flow. It was kept there for 24 h. Total biofilm formed was analyzed by crystal violet (CV) staining as described previously (Ramírez et al., 2015). In short, the liquid content of each well was gently removed with a pipette. Then the wells were washed three times with 900 μl phosphate-buffered saline (PBS, NaCl 8 g/l; KCl 0.2 g/l; Na2HPO4 1.44 g/l; KH2PO4 0.24 g/l; pH 7.4 (Merck)). The final biofilm was stained with 800 μl of 0.1 % (w/v) of CV (Merck) for 30 min at room temperature. The excess CV was removed, and the wells were washed three times with 900 μl PBS with the aid of a micropipette. Finally, retained CV was solubilized in 800 μl 70% ethanol for 45 min and 200 μl of each sample was transferred to a 96-well microplate and the absorbance was measured at 595 nm in a microplate reader (SpectraMax, Molecular Device).

#### 2.4.2 Viable cell counting

Plate counting was used to quantify the number of viable cells in mature biofilms at 24 h (biofilm cells). The medium of the incubated plate was removed via pipetting and the biofilm was washed three times with 900 μl PBS and the resulting biofilm was scraped from the wall of the wells and resuspended in 1ml PBS. The dilution series in PBS were prepared and plated on MRS agar and CFUs were determined after 48 h incubated at 30 °C. Biofilms obtained from the following experiments were exposed to enzyme treatments; static condition experiment (24 h, 30 °C) and the experiment under 3.2 ml/h flow of medium (24 h, 30 °C). In this analysis, first, mature biofilms were washed once with PBS. In the next step, either 1ml of 100 μg/ml of DNase I solution (final concentration in PBS; Sigma-Aldrich), or a solution with 10 μg/ml of Proteinase K (Qiagen), or 1 ml PBS (as a control experiment) was added to the different wells and incubated at 30 °C for 1 h. After the treatment, the wells were washed three times with PBS followed by quantification of residual biofilm using CV staining, whereas, in a parallel experiment, the number of viable biofilm cells was determined by plate counting.

### 2.5 Growth rate and doubling time

Lag time and doubling time for *L. plantarum* CIP104448 and WCFS1 were determined based on plate counting as described previously (Biesta-Peters et al., 2010). The two strains were inoculated to 10 ml De Man-Rogosa-Sharpe (MRS) −80 °C stocks and incubated for 18 h at 30 °C. The 10-fold diluted brain heart infusion supplemented with 0.2% glucose and 0.0005 % manganese sulphate was inoculated with the overnight culture to an initial level of 10^5^ CFU/ml. The culture was incubated at 30 °C, and the number of cells was calculated every 45 min by the plate count method, and the logarithm of CFU/ml was plotted as a function of time to indicate the lag time and growth rate (Supplementary Information, Figure S1). The linear part of the Log-Lin plot represents the logarithmic growth phase of the growth curve, while the lag phase occurred before this phase. The slope of the linear part of the plot determines the growth rate (r) and the doubling time (t_d_) is calculated based on t_d_ = Ln (2)/r (Maier et al. 2010).

### 2.6 Data analysis

All experiments were performed in three independent biological replicates and each replicate includes three technical replicates. The one-way analysis of variance (ANOVA) followed by Duncan test was applied in SPSS to determine whether the flow and initial attachment times had a significant effect on biofilm formation. P < 0.05 was considered as significantly different.

## 3 Results

### 3.1 Mapping the hydrodynamic flow field of the designed flow system and biofilm formation

**Figure 2:**
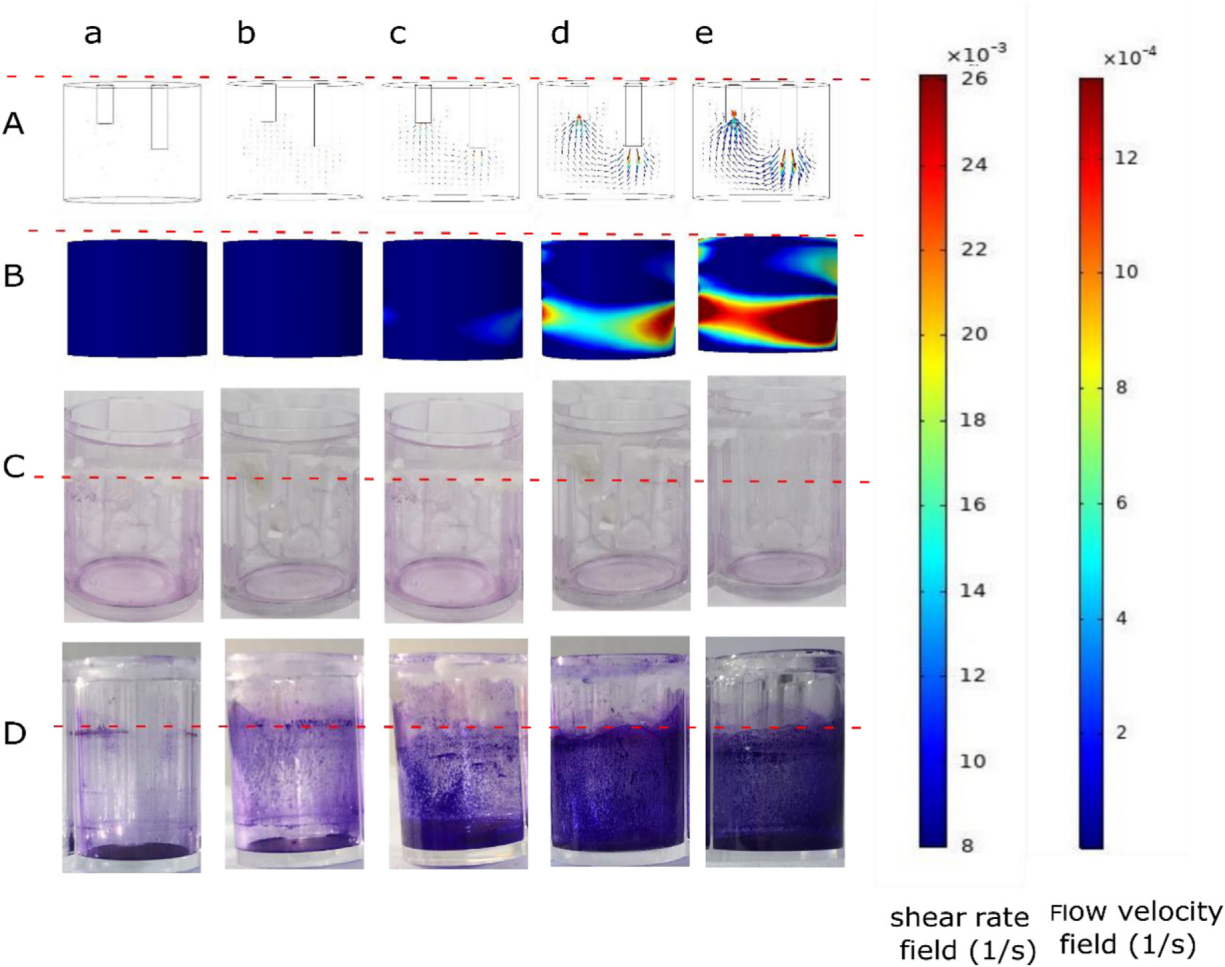
Characteristics of the flow system and biofilm formation in the wells. Row A: Numerical simulation for the cross-section of the velocity field at different flow rates: (a) no flow (b) 0.8 ml/h, (c) 1.6 ml/h, (d) 3.2 ml/h, (e) 4.8 ml/h. Row B: numerical simulation results for shear rate on the wall of the well. The color scale bars represent the shear rate in 1/s and velocity in m/s. Rows (C and D) images of formed biofilms on the wall of a microtiter plate well with WCFS1 and CIP104448 strains, respectively. The horizontal red dashed lines show the level of the culture medium in the wells.

The results of the numerical simulation for the flow velocity profile in the wells (A) and shear rate on the walls of the wells (B) are presented in Figure 2. The images show the cross-section of the velocity field for different flow rates (from a to e). The flow direction and intensity are shown by the arrows. The largest velocities are observed at the inlet and outlet and the minimum velocities are around the corners as expected, and increasing the flow rate caused an increase in the shear rate at the wall of the wells. Rows C and D of Figure 2 show images of CV-stained biofilms formed after 24 h in static and flowing conditions for *Lactobacillus plantarum* strains CIP104448 and WCFS1, respectively. By increasing the flow rate (a to e) the formed biofilm of strain CIP104448 visibly increased. At higher flow rates biofilm formation occurred mostly at the wall of the wells, however, for the static condition most of the biofilm formed at the bottom of the well. This might be related to the higher shear rates near the walls of the wells at higher flow rates, providing more nutrients to the biofilm per unit of time to the wall. In contrast to the CIP104448 strain, no obvious difference was observed in CV-stained biofilms formed by the WCFS1 strain at higher flow rates. In the next sections, a quantitative analysis of biofilm formation using CV staining and plate counting is presented.

### 3.2 Effect of flow rate and initial attachment time on biofilm formation

**Figure 3:**
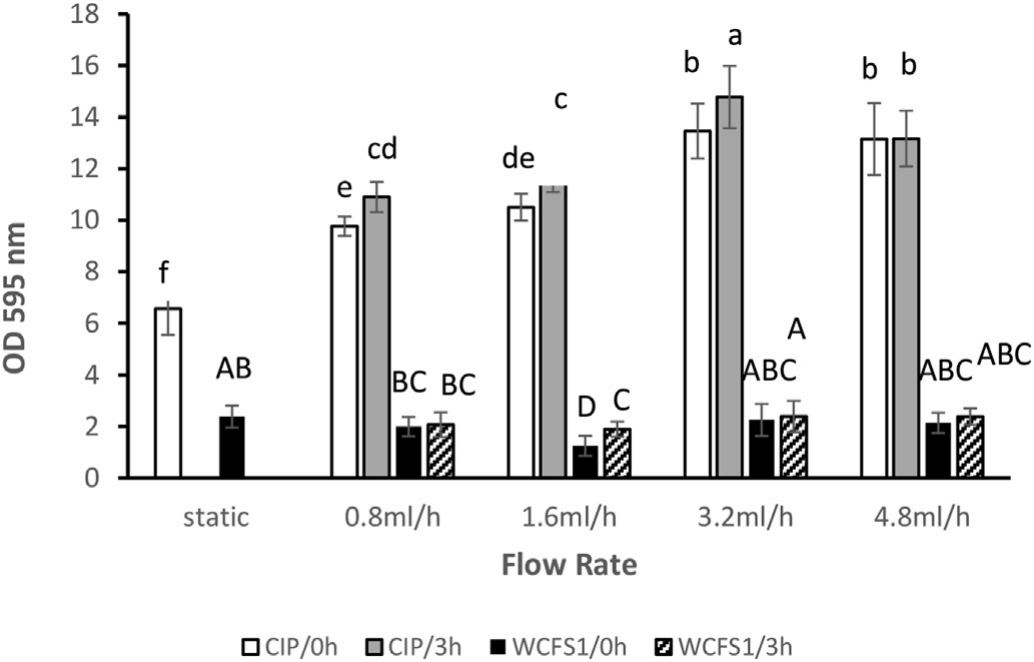
The amount of biofilm formed by *L. plantarum* CIP104448 and WCFS1 as a function of flow rate and pre-incubation time. “Static” represents the flow rate of zero. Biofilms were quantified by CV staining. Different colors and patterns represent different conditions and strains as follows: CIP104448 strain static biofilm and biofilm under flow, with 3 min preincubation time (white bars) and with 3 h preincubation time (grey bars); WCFS1 strain static biofilm and biofilm under flow, with 3 min preincubation (black bars) and with 3 h preincubation time (pattern bars). Letters indicate the results of the Duncan test with a significant difference at p < 0.05. Same letters mean no significant difference.

The amount of biofilm formed by the two *L. plantarum* strains under different flow rates; (0.8, 1.6 ml/h, 3.2 and 4.8 ml/h), after 24 h, was determined for two initial attachment times of 3 min and 3 h and compared with static biofilm formation. Figure 3 shows the amount of CV-stained biofilm material as a function of flow rate for CIP104448 and WCFS1. For the CIP104448 strain, static biofilm formation resulted in an OD 595 of 6.5, while CV staining increased under flowing conditions for the two initial attachment times.

Overall, the amount of biofilm formed by the CIP104448 strain with 3 h attachment time for all flow rates was significantly higher than 3 min initial attachment time except for the flow rate of 4.8 ml/h. For this flow rate, the amount of biofilm was almost equal for both attachment times. Unlike the CIP104448 strain, the amount of the formed biofilm by WCFS1 under flow remained equal to that under the static condition, for all flow rates except for the flow rate of 1.6 ml/h, at which a slight decline was observed in the biofilm formation. The influence of pre-incubation was only significant at a flow rate of 1.6 ml/h where more biofilm was formed with 3 h of initial attachment time compared to the 3 min attachment time. Overall, CIP104448 strain CV staining results showed that the amount of the formed biofilm at 0.8, 1.6, 3.2 and 4.8 ml/h flow rates were approximately 1.6, 1.7, 2.2 and 2 fold higher than in the static condition. Quantification of CV staining data of *L. plantarum* CIP104448 and WCFS1 matches the images of CV-stained wells presented in Figure 2. The amount of formed biofilm by CIP104448 strain in the static condition was approximately 3 fold higher than the biofilm produced by WCFS1 strain under static conditions and this difference increased to more than 6 fold at flow rates of 3.2 ml/h (referring to 3 h pre-incubation conditions).

### 3.3 Quantification of viable biofilm cells under static and constant flow conditions

**Figure 4:**
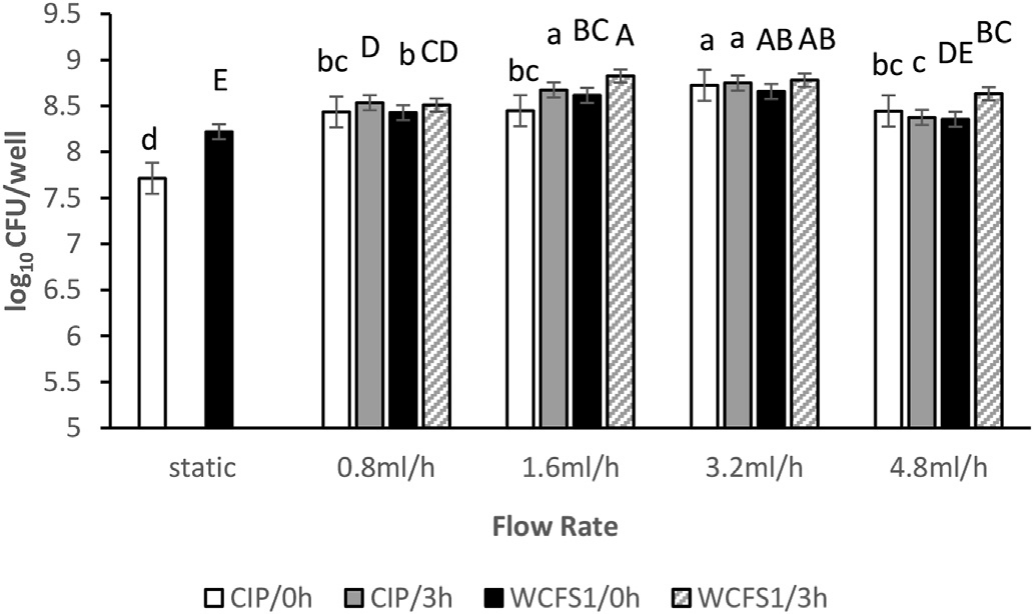
The number of viable biofilm cells of *L. plantarum* CIP104448 and WCFS1 as a function of flow rate and pre-incubation time. These numbers were quantified by plate counting. “Static” represents the flow rate of zero. Different colors and patterns represent different conditions as follows: CIP104448 strain static biofilm and biofilm under flow, with 3 min preincubation time (white bars) and 3 h preincubation time (grey bars); WCFS1 strain static biofilm and biofilm under flow, with 3 min preincubation (black bars) and 3 h preincubation time (pattern bars). Letters indicate the results of the Duncan test with a significant difference at p < 0.05. Same letters mean no significant difference.

For the CIP104448 strain, the number of viable biofilm cells increased under constant flow conditions (Figure 4). Under static conditions, mature biofilms, after 24 h, contained 7.7 log_10_ CFUs/well, and biofilm CFUs were higher under the flow condition, with the highest cell counts (8.7 log10 CFUs/well) at 3.2 ml/h. The only influence of preincubation time on the number of viable cells in mature biofilm was observed at 1.6 ml/h, where more viable cells were observed for the 3 h preincubation condition (p < 0.05).

*L. plantarum* WCFS1 showed a similar behaviour; biofilm cell counts increased from 8.2 log_10_ CFUs/well in static condition to 8.8 log_10_ CFUs/well under flow condition (Figure 4). No significant differences were observed between biofilm cells counts when comparing the 3 min and 3 h preincubations.

### 3.4 Correlation between biofilm CV staining and biofilm cell counts

**Figure 5:**
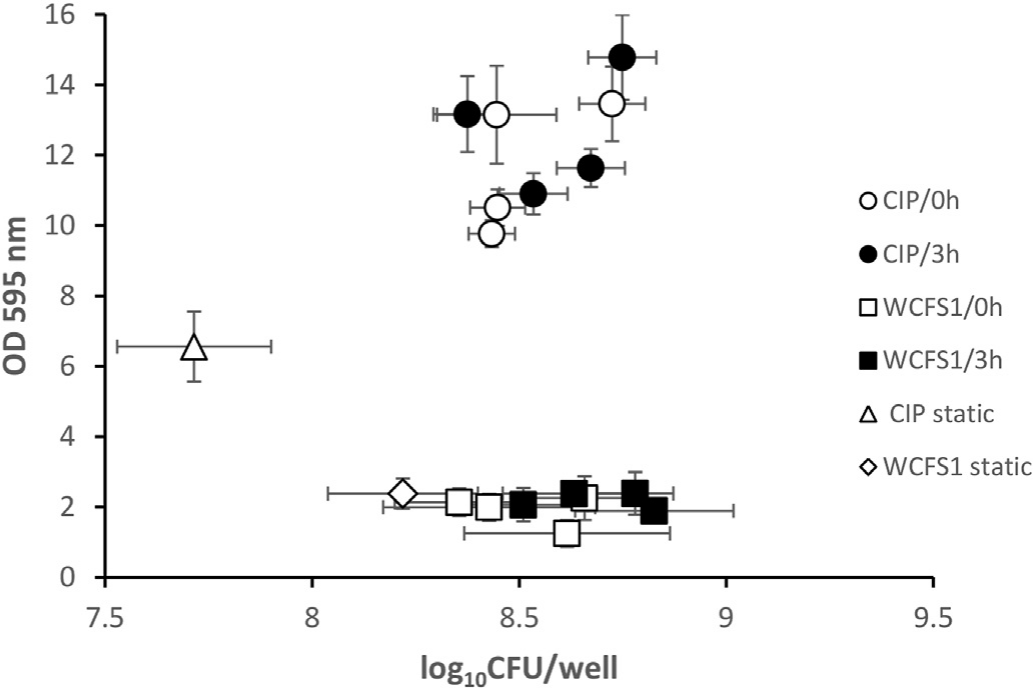
Correlation between biofilm cell counts and biofilm CV staining of *L. plantarum* CIP104448 and WCFS1. The plotted data includes the points obtained for each strain under static conditions, and under 4 different flow rates, with 3 min and 3 h preincubation times (9 data points for each strain). Different symbols represent different experiments: strain CIP104448 with 3 min (open circles) and 3 h preincubation (closed circles), strain WCFS1 with 3 min (open squares) and 3 h preincubation (closed squares). Strains CIP104448 (open triangles) and WCFS1 (open diamonds) in static conditions.

Statistical analyses of the data in Figures 3 and 4, and the scatter plot of log_10_ CFUs/well as a function of the OD 595nm (CV assay) (Figure 5) indicated that there was a poor correlation between the above-mentioned parameters for the biofilm formed by WCFS1. The correlation between the amount of biofilm formed and biofilm cell counts in CIP104448 strain with 3 min and 3 h preincubation times was 0.734 and 0.840, respectively (Figure 5).

### 3.5 Enzymatic treatment with DNase I and proteinase K

**Figure 6:**
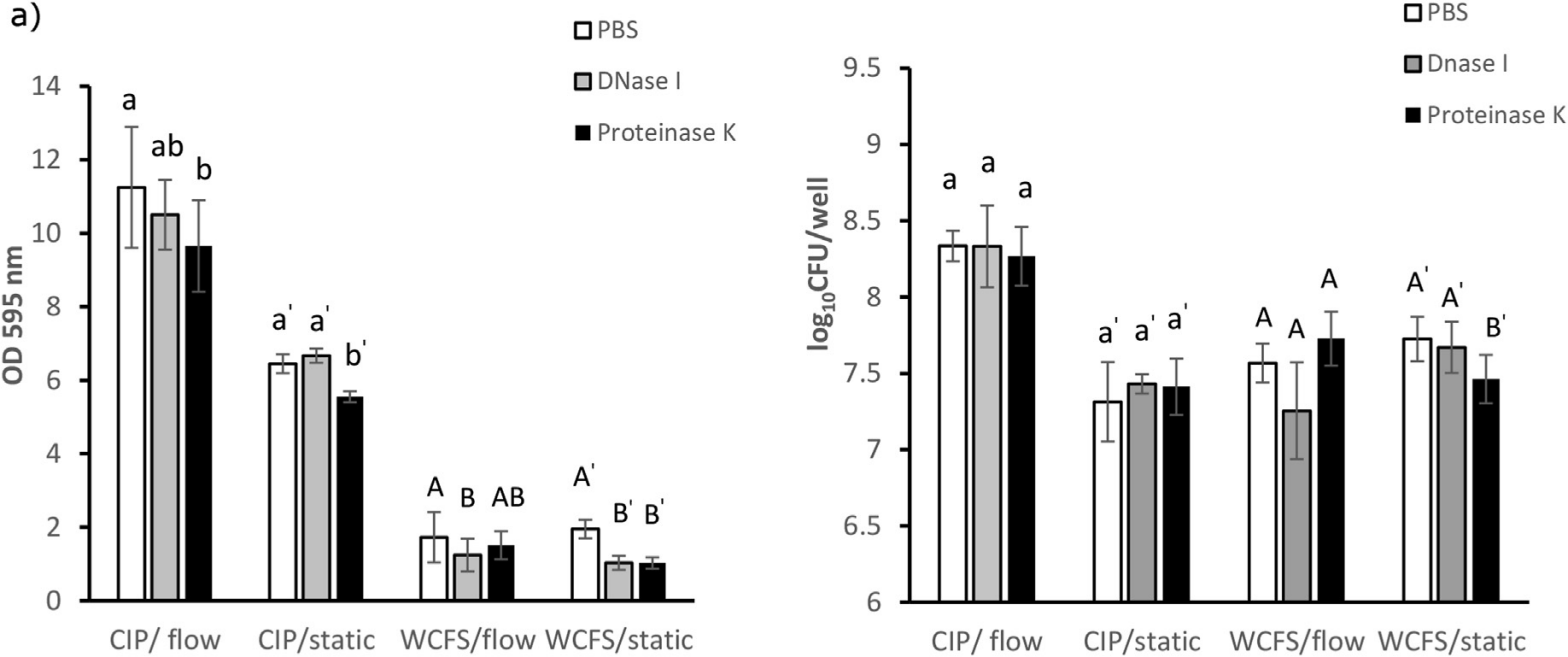
Effect of DNase and Proteinase K treatment on mature biofilms formed in static and under flow (3.2 ml/h) conditions by *L. plantarum* CIP104448 and WCFS1. (a) Amount of biofilms was quantified by CV staining. (b) Number of viable cells. The graph presents the mature biofilms treated with PBS (control, white bars), 100 μg/ml DNase I (grey bars) and 10 μg/ml Proteinase K (black bars). Letters indicate the results of the Duncan test with a significant difference at p < 0.05. Same letters mean no significant difference.

Mature biofilms obtained from both dynamic and static flow conditions were treated with DNase I and Proteinase K to identify the presence of extracellular DNA (eDNA) and protein components in the biofilm structure. Figure 6 shows the amount of biofilm and the number of viable cells as a function of enzyme treatment on mature biofilms of CIP104448 and WCFS1 strains. For *L. plantarum* CIP104448, the amount of biofilm stained by CV decreased only after the addition of Proteinase K, while DNase I treatment did not have a significant effect on biofilms formed in static and flowing conditions. Notably, both DNase I and Proteinase K caused a significant decline in the amount of CV stained biofilm formed by WCFS1 in the static condition, while biofilm formed under the flowing conditions was only sensitive to DNase I. Quantification of viable biofilm cells after enzyme treatments (Figure 6b), showed that neither DNase I nor Proteinase K had a significant effect on biofilm cell counts of CIP104448, both in static and under flow conditions and also on WCFS1 biofilm under flow conditions. However, the effect of proteinase K treatment on WCFS1 biofilm formation in the static condition was significant while the DNase I treatment did not result in a significant reduction.

### 3.6 Cell growth in time

**Figure 7:**
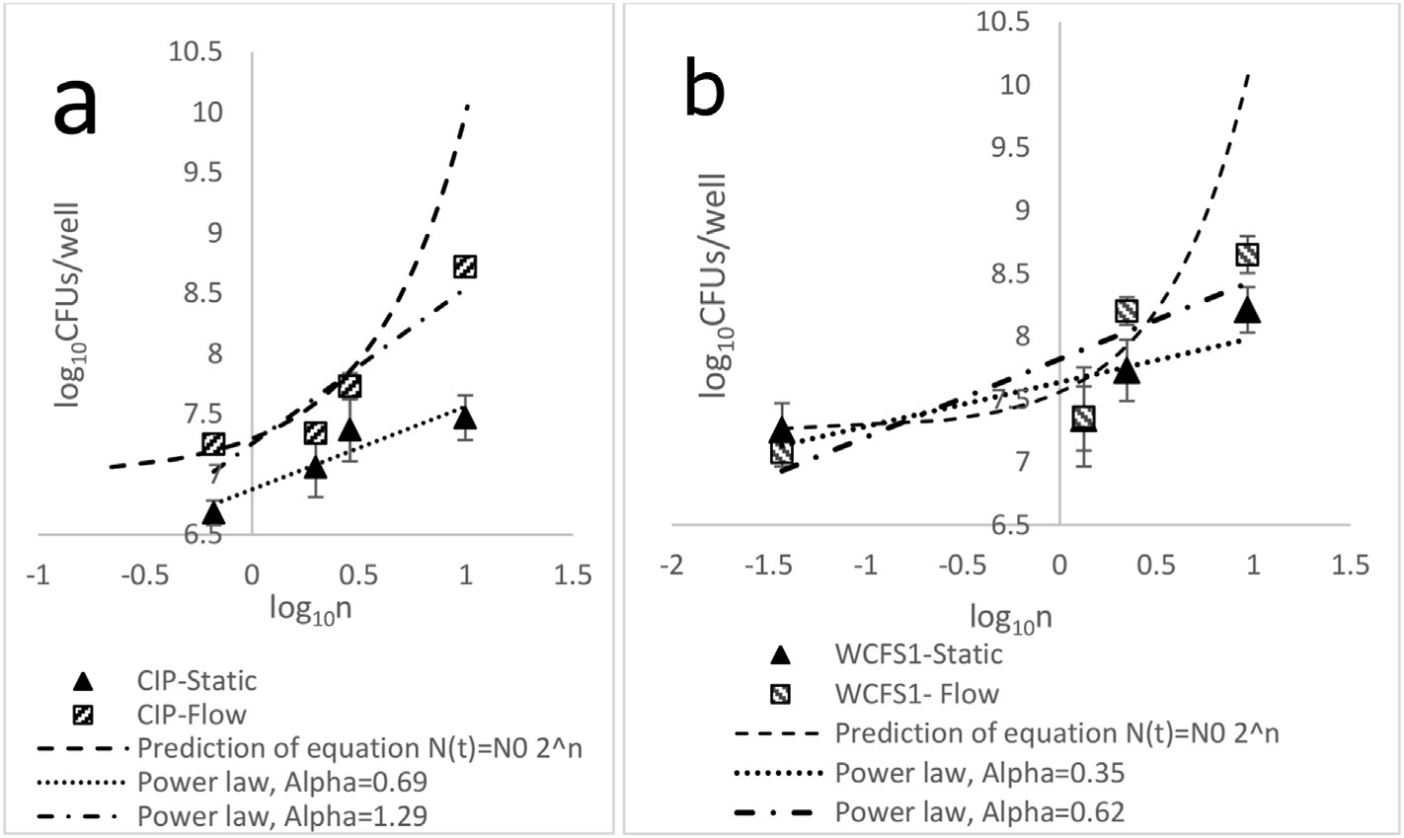
Logarithm of the number of viable biofilm cells of *L. plantarum* CIP104448 (a) and WCFS1 (b) as a function of logarithm of rescaled time (n) where *n* = (*t* − *t*_*1*_)/*t*_*d*_. Patterned squares and filed triangles are the experimental results for the logarithm of the number of viable bacteria in the biofilm formed under flow with a flow rate of 3.2 ml/h, and static condition, respectively. The Dash-dotted line shows the power-law fit to under flow experiments for CIP104448 and WCFS1 with exponents of 1.29 and 0.62, respectively. Dotted lines show the fitted power-laws with exponents 0.69 and 0.35 for CIP104448 and WCFS1 respectively in the static conditions. Dashed lines, show the prediction of equation *N*(*t*) *= N*_*0*_*2*^*n*^.

To predict the number of viable biofilm cells, first, the lag time (t_1_) and doubling time (t_d_) of the two strains were determined as described in the method section and SI. The lag time was 90 and 180 min for WCFS1 and CIP104448, respectively and the doubling times was about 136 min for both strains (data are shown in SI). In theory, after each doubling time, the number of cells increases two-fold, therefore, the number of cells in time reads as *N*(*t*) = *N*_*0*_ *2*^*n*^, where $n=\left(t-t_{l}\right)/t_{d}$ is the number of cell divisions in which the population doubles in size, and *N*_*0*_ is the initial number of cells that are attached to the surface (Dalgaard and Koutsoumanis 2001). We have quantified experimentally the number of viable bacteria attached to the surface of the well, at the initial stage (*N*_*0*_) and after 3, 6, 8 and 24 h for both static and under flow conditions (flow rate of 3.2 ml/h). In addition, we can theoretically estimate the evolution of the number of bacteria in the biofilm as a function of time from the above equation with the parameter *N*_*0*_ being 1.1∗10^6^ CFUs and 1.7∗10^6^ CFUs for CIP104448 and WCFS1, respectively, as determined by the plate count experiments. In the above theoretical equation, it is assumed that all bacteria remain alive and contribute to the doubling process and remain in the biofilm. Figure 7 shows the logarithm of the number of cells as a function of the logarithm of the number of cell divisions (*Log* (*n*)). The prediction of the above equation for the number of cells, and the experimental data are presented for CIP104448 (Figure 7 a) and WCFS1 (Figure 7 b) for the flow rate of 3.2 ml/h and static conditions in Figure 7. As it is clear from Figure 7, the prediction of *N*(*t*) *= N*_*0*_ *2*^*n*^ for biofilm cell growth (shown by dashed line) is significantly higher than the experimental values in the course of the experiment, conceivably due to inhibition of growth, cell death and/or dispersal of cells from the biofilm. Intrestingly the experimental data for both strains roughly show a linear trend in a Log-Log scale which indicates a power-law relation between the number of cells and number of doubling $as\;({N\left(t\right)∼((t-t_{l})/t_{d})}^{\alpha }$), where α is a fitting parameter. Fitted power-law relations to the experimental data show a good agreement. Dashed-doted lines are fitted to under flow data while dotted lines are fitted to static ones. The fitted exponent α is reported in Table 1 for each experiment.

**Table 1:** Exponent α obtained by fitting power-law equation to experimental data of Figure 7. Higher values of α indicate more rapid growth of the number of cells.

Strain	Condition	α
CIP104448	Flow	1.29
CIP104448	Static	0.69
WCFS1	Flow	0.62
WCFS1	Static	0.35

## 4 Discussion

In many natural and technological processes, microbial biofilms are formed and grown under fluid flow. The presence of flow strongly influences different stages of biofilm formation, from attachment to disruption (Thomen et al., 2017). On the one hand, the flow affects the movement of bacteria and the formation of biofilms by applying mechanical force to them. On the other hand, flow changes the metabolic rate and gene expression of bacteria due to altering the distribution of nutrients and oxygen and washing away chemical signals (Emge et al., 2016). In this study, we designed a device based on a conventional microtiter plate to study the effect of flow on biofilm formation by two strains of *Lactobacillus plantarum*.

Unlike previous approaches for studying biofilm formation under flow, we used the microtiter plate as the container in our device which enables us to quantify biofilm formation under flow based on crystal violet staining and plate counting that are typically applied in microbiological analyses and comparing them with the result of the static condition.

As indicated in the results section we found that applying flow significantly influenced biofilm formation by the *L. plantarum* CIP104448 strain and that the total amount of the formed biofilm grew when increasing the flow rate. This increase of the total biofilm quantified using CV staining could be the outcome of increasing the matrix component and/or the number of cells (dead and alive). For finding the correlation between the amount of the total biofilm and viable cells we also measured the number of viable cells. On the one hand, applying the flow causes the shear trapping phenomenon (Rusconi et al. 2014), which causes bacteria to move to high shear regions that are near the walls and increases the probability of attachment of cells to the surface which is the first step in biofilm formation. Therefore, the number of bacteria in the biofilm increased as presented in Figure 4. On the other hand, the flow of medium transfers nutrients to the bacteria in the biofilm. The availability of nutrients stimulates the growth and reproduction of bacteria as shown previously for *E. coli* (Teodósio et al., 2011). In addition, shear stress can induce extracellular polymeric substances (EPS) formation in some species, which protects the biofilm from detachment under the flow (Park et al., 2011). Therefore, the increase in biofilm amount can occur up to an optimal flow rate, and further increase of the flow may cause detachment of cells and biofilm and consequently a decline in the amount of biofilm as observed for the CIP104448 strain, where a decline happened at the highest flow rate of 4.8 ml/h. The detached biofilms can also contain viable cells. The same phenomenon was observed by Park et al. for biofilms formed by *Pseudomonas aeruginosa* in a microfluidic system (Park et al., 2011). Increase in the preincubation time can slightly improve the biofilm formation for the CIP104448 strain. This could be attributed to longer residence times of cells on the surfaces, which provides them a higher chance of attachment to the surface and subsequent increase of biofilm formation. This may not be a generic mechanism that applies to *L. plantarum* strains in general, since it was observed that the amount of formed biofilm did not significantly change under flow conditions for the WCFS1 strain.

Measuring the number of viable cells showed that biofilm cell counts increased under flow conditions with respect to the static condition for both strains. However, Figure 5 shows that the correlation between the number of bacteria and the amount of formed biofilm was poor for the WCFS1 strain, as previously reported for biofilm formation under static conditions (Ramírez et al., 2015). This implies that CV staining does not provide a good estimate for the number of bacteria. This is understandable, since CV may also bind dead cells, proteins and other cellular components. Cell death could result in cell lysis and the release of eDNA that increases the CV staining (Ramírez et al., 2018).

Furthermore, in Figure 2, for the CIP104448 strain under static conditions, the biofilm was formed at the bottom of the wells while applying the flow induced biofilm formation on the walls of the wells as well. In static conditions, biofilm formation is mostly due to the sedimentation of non-motile bacteria such as *L. plantarum*, at the bottom of the wells. However, for biofilm formation under flow, besides the sedimentation, the shear trapping phenomenon causes the formation of biofilm on vertical solid surfaces (Rusconi et al., 2014). Our observation in sequence images in Figure 2 for the CIP104448 strain, confirms to some extent the above-mentioned claim. Figure 2 also shows the shear rate at wall grows by increasing the flow rate. We note that the pattern of CV staining in the third row does not map with the field of shear rate in the second row; according to numerical analyses, the shear rate in red parts is higher than in the blue parts, whereas the CV staining patterns uniformly distributed all over the wall. However, the formed biofilm on the walls could have a nonuniform profile of thickness that matches the shear rate profile. To explore the thickness profile of the formed biofilm in detail, an advanced assay is needed which is beyond the scope of this paper.

It is known that extracellular polymeric substances play an important role in constructing the 3D structure of a biofilm and the 3D structure may contain also eDNA, protein, and/or polysaccharides. In the CIP104448 strain, biofilms formed under static and flow conditions showed no effect of DNase I treatment while following Proteinase K treatments, reduction of staining was observed, which may suggest the presence of protein and/or proteinaceous material in the structure of the biofilm. The fact that no change in the amount of biofilm after DNase I treatment was observed, could be due to the lack of eDNA in the structure or eDNA in the biofilm not being accessible by the enzyme. For the WCFS1 strain, both DNase I and Proteinase K treatments caused a decline in the amount of mature biofilm in the static condition and suggested the contribution of both proteins/proteinaceous material and eDNA to the biofilm matrix, in line with previous observations (Ramírez et al., 2015). However, under the flow condition, the biofilm formed by the WCFS1 strain was only sensitive to DNase I. This means that the presence/accessibility of proteins and/or proteinaceous material in the biofilm formed by WCSF1 strain was different in static and dynamic conditions. Therefore, flow conditions can affect the composition and structure of the biofilm formed by this strain.

On the one hand, under static conditions, bacteria have a chance to sediment and stick to the bottom of the well or each other and both contribute to biofilm formation. On the other hand, under dynamic conditions, bacteria can be moved by the flow and could attach to the vertical surfaces where they have access to high nutrient levels all the time. Our results show that the growth of the number of bacteria over a considerably long period does not obey the simple theoretical prediction of ${N\left(t\right)∼2}^{n}$. It is mainly due to the existence of a fraction of bacteria that do not contribute to the doubling process because of several factors such as growth arrest, mortality, and/or dispersion of biofilm cells. The growth of the number of bacteria in time is best fitted with a power-law ${N\left(t\right)∼\left(n\right)}^{\alpha }$ equation with the growth exponent α depending on the flow conditions. The exponent obtained by fitting the experimental results is higher for the dynamics conditions with respect to the static ones for both strains, in line with the presumed growth stimulation by the continuous supply of nutrients. In addition, the CIP104448 strain shows higher exponents with respect to WCFS1 for both conditions. The power-law behaviour observed here may provide insights for the predictions of biofilm cell growth behaviour and its underlying mechanism in future research.

In summary, we introduced a novel fluidic assay to investigate biofilm formation under flow conditions using non-motile *L*. *plantarum* strains CIP104448 and WCFS1 and compared our results with static conditions. We used CV staining and plate count experiments for quantifying the formed biofilms and the presence of viable biofilm cells. Quantification of DNase and protease K treatments on these parameters revealed differences in biofilm resilience between the tested *L. plantarum* strains and respective biofilms formed under flow and static conditions. We have also simulated the flow field of the medium to determine the velocity and shear fields and compared them to the spatial distribution of formed biofilms. Besides, we have found that the number of cells attached to the wall follows a power-law behaviour in time. The combined results show the advantages of the new fluidic system including the option to combine microbiology and physics for quantitative analysis of the impact of a range of parameters including medium composition, temperature, and flow rate on microbial biofilm formation and biofilm matrix characteristics.

## Declarations

### Author contribution statement

Mehdi Habibi: conceived and designed the experiments; analyzed the data; discussed the experimental results and analyses; wrote the main part of the manuscript; editing the manuscript and interpreting the data.

Parisa Rashtchi: conceived and designed the experiments; performed the experiments; analyzed the data; discussed the experimental results and analyses; wrote the main part of the manuscript; editing the manuscript and interpreting the data.

Marcel Tempelaars: conceived and designed the experiments; performed the experiments; analyzed the data; discussed the experimental results and analyses; editing the manuscript and interpreting the data.

Erik van der Linden: conceived and designed the experiments; discussed the experimental results and analyses; editing the manuscript and interpreting the data.

Tjakko Abee: conceived and designed the experiments; analyzed the data; discussed the experimental results and analyses; editing the manuscript and interpreting the data.

### Funding statement

This research did not receive any specific grant from funding agencies in the public, commercial, or not-for-profit sectors.

### Data availability statement

Data will be made available on request.

### Declaration of interest's statement

The authors declare the following conflict of interests: The authors declare no competing interests.

M. H. is a member of the editorial board of the journal of Applied Rheology, and the editorial board of Applied Mechanics Journal (MDPI). T. A. is acting as associated editor of Microbiology, a journal published by the Society for General Microbiology (UK).

### Additional information

Supplementary content related to this article has been published online at https://doi.org/10.1016/j.heliyon.2022.e12602.

## Appendix A Supplementary data

SI Rashtchi et al.docx SI Rashtchi et al.docx
