# Supplementary material for: Lactobacillus plantarum strains show diversity in biofilm formation under flow conditions
Source: Heliyon. 2022 Dec 24;8(12):e12602. doi: 10.1016/j.heliyon.2022.e12602 (PMC9816783; doi:10.1016/j.heliyon.2022.e12602)
Supplement: SI Rashtchi et al.docx [file mmc1.docx]

**Supplementary Information**

***Lactobacillus plantarum* strains show diversity in biofilm formation under flow conditions**

P. Rashtchi ^1,2^, M. Tempelaars ^2^, E. van der Linden ^1^, T. Abee ^2^, M. Habibi ^1*^

*1)Physics and Physical Chemistry of Foods, Wageningen University, Wageningen 6708WG, The Netherlands*

*2) Food Microbiology, Wageningen University, Wageningen 6708WG, The Netherlands*

**: Corresponding author*

*Email address:* [*mehdi.habibi@wur.nl*](mailto:mehdi.habibi@wur.nl)

**Calculating lag and doubling times:**

We calculated the lag time and doubling time, based on viable cell plate count measurement. Fig 1S shows the logarithm of the number of cells (log_10_ CFU/ml) as a function of time. The linear part of the plot represents the logarithmic growth phase, and before that, the lag phase occurs. After the logarithmic growth phase, the growth rate decreases and we arrive at a cross-over regime; therefore, only data points shown in the red ovals are considered to calculate the growth rate and doubling time. The slope of the linear part is the growth rate (r). Doubling time is calculated by the t_d_=Ln(2)/r equation.

b)

a)

Fig S1: Evolution of logarithm of the number of cells in time at 30°C in a 10-fold diluted brain heart infusion supplemented with 0.2% glucose and 0.0005 % manganese sulfates for two Lactobacillus plantarum strains: a) CIP104448 strain b) WCFS1 strain. The vertical dashed lines represent the lag time (t_l_).

Table S1: Lag time and doubling time for CIP104448 and WCFS1 strain based on data in Fig S1.

| Kinetical Parameters | CIP104448 | WCFS1 |
| --- | --- | --- |
| Lag time | 90 min | 180 min |
| Doubling Time | 136 min | 136 min |

**Effect of DNase I and Proteinase K**

Table S2: The effect of DNase I and Proteinase K on mature biofilm formed in 48 well microplates.

| Strain | Flow condition | Enzyme treatment | | |
| --- | --- | --- | --- | --- |
|  |  | PBS( control) | DNase I | Proteinase K |
| CIP104448 | Flow | 8.33 | 8.33 | 8.26 |
| CIP104448 | Static | 7.31 | 7.43 | 7.41 |
| WCFS1 | Flow | 7.56 | 7.25 | 7.72 |
| WCFS1 | Static | 7.72 | 7.67 | 7.46 |
